# Bis(2,3-dibromo-4,5-dihydroxybenzyl) Ether, a Marine Algae Derived Bromophenol, Inhibits the Growth of *Botrytis cinerea* and Interacts with DNA Molecules

**DOI:** 10.3390/md12073838

**Published:** 2014-06-27

**Authors:** Ming Liu, Genzhu Wang, Lin Xiao, Xuanli Xu, Xiaohui Liu, Pingxiang Xu, Xiukun Lin

**Affiliations:** 1 Key Laboratory of Marine Drugs, Ministry of Education, School of Medicine and Pharmacy, Ocean University of China, Qingdao 266003, China; E-Mails: lmouc@hotmail.com (M.L.); 15133634098@163.com (G.W.); 2 College of Chemistry and Pharmaceutical Sciences, Qingdao Agricultural University, Qingdao 266109, China; 3 Department of Pharmacology, Capital Medical University, Beijing 100069, China; E-Mails: xhl52@msn.com (X.X.); xiukunlin@126.com (X.L.); xupingxiang@163.com (P.X.)

**Keywords:** bis(2,3-dibromo-4,5-dihydroxybenzyl) ether, *Botrytis cinerea*, antifungal, DNA interaction

## Abstract

Bis(2,3-dibromo-4,5-dihydroxybenzyl) ether (BDDE) is a bromophenol isolated from marine algae. Previous reports have shown that BDDE possesses cytotoxic and antibacterial activity. In the present study, we demonstrate that BDDE displays broad-spectrum antifungal activities, especially on *Botrytis cinerea*. BDDE inhibits the growth of *B. cinerea* cultured on a solid medium of potato dextrose agar (PDA) as well as on the potato dextrose broth (PDB) medium. Moreover, BDDE decreases the incidence of fruit decay and severity of strawberries infected with *B. cinerea*. Further studies have revealed that BDDE decreases the germination rate and inhibits the mycelial growth of *B. cinerea*. The inhibition mechanisms are related to the disruption of the cell membrane integrity in *B. cinerea* spores and newly formed germ tubes. This study also suggests that BDDE possibly interacts with DNA via intercalation and minor groove binding. The studies provide evidence that BDDE has potential application in the control of gray mold after fruit harvest and the compound could serve as a candidate or lead template for rational drug design and for the development of antifungal agents.

## 1. Introduction

Phytopathogenic fungi, one type of major parasitic organisms, constitute a main threat to plants, and usually induce serious diseases and yield losses in crops [1]. Currently, infection by phytopathogenic fungi on plants, such as gray mold, is generally treated with synthetic fungicides to control susceptible pathogens [2]. Although the synthetic fungicides are effective and used widely, continuous application of these fungicides has resulted in loss of biological control and has led to drug resistance and environmental problems [3]. Moreover, synthetic fungicides often induce concern in food safety. To overcome these problems, continuous efforts to find safer, more effective control options with minimal risk to human health and the environment is urgent.

Marine bromophenols, usually existing in marine sponges and algae, have attracted much attention in the field of antimicrobial agents [4]. Accumulated evidence indicates that marine bromophenols possess promising antibacterial activity [5,6,7] and antiviral activities [8,9]. For example, a synthesized bromophenol compound, 2,4,6,2′,4′,6′-hexabromodiorcinol, shows potent antibacterial activity against several pathogenic bacteria with MIC values ranging from 0.556 to 1.11 μM [7]. In addition, symphyocladin G, a new bromophenol adduct derived from marine red alga *Symphyocladia latiuscula*, is found to possess antifungal activity against *Candida albicans* [10]. Several bromophenols isolated from red alga *Odonthalia corymbifera* have been reported to be promising candidates for antifungal agents in crop protection. These bromophenols could inhibit the pathogenicity of fungus *Magnaporthe grisea* and reduce the appressorium formation on rice plants [11].

**Figure 1:**
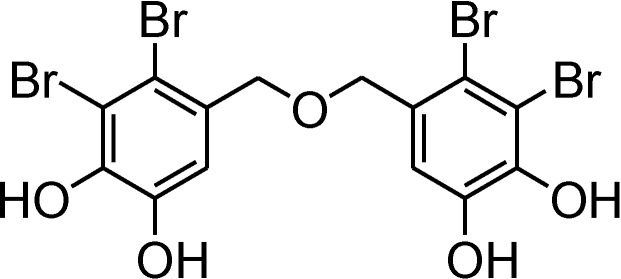
Chemical structure of bis(2,3-dibromo-4,5-dihydroxybenzyl) ether (BDDE).

Bis(2,3-dibromo-4,5-dihydroxybenzyl) ether (BDDE, Figure 1A), isolated from the marine algae *Leathesia nana*, *Rhodomela confervoides*, and *Rhodomela confervoides*, possesses a variety of bioactivities, such as cytotoxicity to cancer cells [12,13], inhibition of protein tyrosine phosphatase 1B [14], and α-gulcosidase [15,16,17,18]. BDDE also exhibits antibacterial activity against several strains of Gram positive and Gram negative bacteria [6]. In this study, we demonstrate that BDDE displays antifungal activities on several strains of fungal pathogens, and has the potential to control gray mold in fruit caused by *Botrytis cinerea*. We find that BDDE inhibits the spore germination and germ tube elongation of *B. cinerea*, and the mechanisms are related to the disruption of cell membranes in *B. cinerea* and the interaction with DNA.

## 2. Results

### 2.1. BDDE Inhibits the Mycelial Growth of Fungal Pathogens

**Figure 2:**
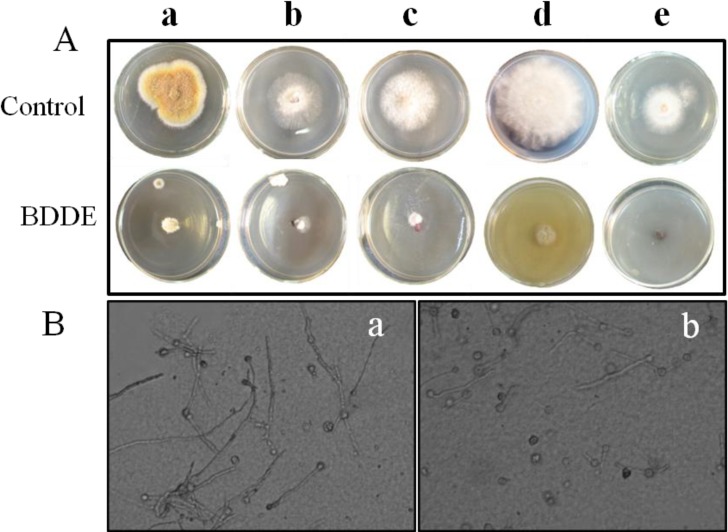
BDDE inhibits the mycelial growth of fungal pathogens. (**A**) The inhibitory effect of BDDE on the mycelial growth of fungal pathogens. The fungal pathogens including *B. Cinerea* (**a**); *Valsa mali* (**b**); *Fusarium graminearum* (**c**); *Coniothyrium diplodiella* (**d**); *Colletotrichum gloeosporioides* (**e**) were cultured on potato dextrose agar (PDA) medium and treated with BDDE for 4, 2, 3, 2, 3 days, respectively. Three replicates were performed for each fungus; (**B**) BDDE inhibits the mycelial growth of *B. cinerea* cultured in potato dextrose broth (PDB) liquid medium. Fungal spores were pre-germinated in PDB medium for 24 h. Then the spores with germ tube were incubated for another 24 h in the absence (**a**) and presence (**b**) of 100 μg/mL BDDE.

To evaluate the antifungal activities of BDDE *in vitro*, we examined its inhibition on mycelial growth to seven fungal pathogens. As shown in Table 1 and Figure 2A, BDDE (100 μg/mL) displayed broad and potent inhibition on the mycelial growth of *B. cinerea* (a), *Valsa mali* (b), *Fusarium graminearum* (c), *Coniothyrium diplodiella* (d), and *Colletotrichum gloeosporioides* (e), but no inhibition on *Alternaria mali Roberts* and *Alternaria porri* (Table 1). Among these pathogens, *B. cinerea* was relatively more sensitive to BDDE with an inhibition rate of about 83.3% (Table 1). BDDE caused obvious decreases in the colony expansion cultured on PDA medium plate (Figure 2Aa). In addition, BDDE could also inhibit the mycelial growth of *B. cinerea* in PDB medium (Figure 2B). Therefore, *B. cinerea* was selected for further analysis.

**Table 1:** Antifungal activities of BDDE against seven fungal pathogens on PDA medium plate containing 100 μg/mL BDDE. Three replicates were used for each fungus.

Pathogenic Fungi	Inhibition Rate (%)
*Botrytis cinerea*	83.3 ± 6.8
*Valsa mali*	80.0 ± 7.2
*Fusarium graminearum*	77.1 ± 5.3
*Coniothyrium diplodiella*	75.0 ± 8.5
*Colletotrichum gloeosporioides*	67.7 ± 5.9
*Alternaria mali Roberts*	0
*Alternaria porri*	0

### 2.2. BDDE Inhibits the Growth of Gray Mold on Strawberries

**Figure 3:**
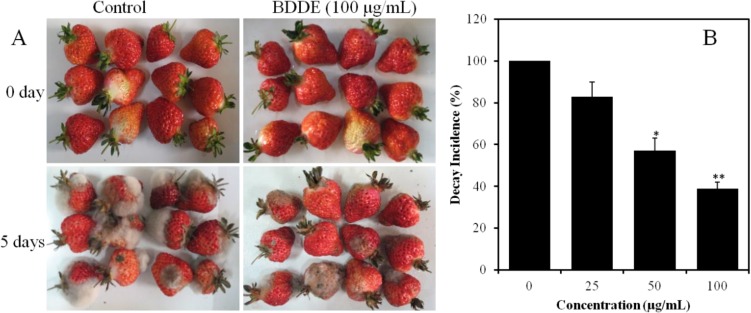
Inhibitory effect of BDDE on strawberry gray mold caused by *B. cinerea*. (**A**) Representative pictures of strawberries treated with (100 μg/mL) or without BDDE for 5 days at 23 °C; (**B**) Histogram shows the decay incidence in the absence or presence of BDDE. The experiment was repeated three times. Values are expressed as means ± SD. * *p* < 0.05, ** *p* < 0.01 *versus* control indicates significant difference according to Student’s *t*-test.

To further confirm the antifungal activities of BDDE, fruit decay tests were carried out on freshly harvested strawberries. As shown in Figure 3A, the fruits infected with *B. cinerea* for 5 days showed serious decay in the control group. However, the formation of gray mold was delayed and the decay incidence decreased to 83%, 57%, and 39% when treated with BDDE at a concentration of 25, 50, 100 μg/mL, respectively (Figure 3B). These results confirmed that BDDE could inhibit the formation of gray mold on strawberries induced by *B. cinerea*. 

### 2.3. BDDE Inhibits Spore Germination and Germ Tube Elongation of B. cinerea

**Figure 4:**
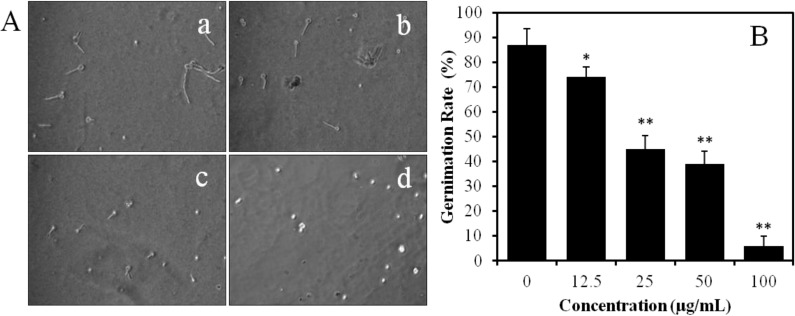
Effect of BDDE on spore germination and germ tube elongation of *B. cinerea* in PDB. (**A**) Spores were treated without (**a**) or with BDDE at a concentration of 25 (**b**), 50 (**c**), and 100 μg/mL (**d**). The germination rate and germ tube elongation were observed using a microscope; (**B**) Histogram shows the germination inhibition rate in the absence or presence of BDDE. Values are expressed as means ± SD. * *p* < 0.05, ** *p* < 0.01 *versus* control indicates significant difference according to Student’s *t*-test.

The effect of BDDE on spore germination and germ tube elongation in PDB medium was investigated. As shown in Figure 4A, the spore germination of *B. cinerea* was significantly inhibited by BDDE in a concentration dependent manner. The germination rate was 87% in the control group. However, the rate was decreased significantly when the spores were treated with BDDE; the germination rate decreased to 74%, 45%, 39%, and 6%, when treated the spores with 12.5, 25, 50, and 100 μg/mL BDDE, respectively (Figure 4B). The IC_50_ value of BDDE on *B. cinerea* germination is about 31 μg/mL. In addition, BDDE also suppressed the elongation of germ tube. Germ tube elongation decreased with the increasing concentration of BDDE, and was almost completely inhibited by BDDE at 100 μg/mL (Figure 4A). These results indicated that both spore germination and germ tube elongation were inhibited by BDDE.

### 2.4. BDDE Destroys the Membrane Integrity of B. cinerea

In order to illustrate the mechanisms underlying the BDDE inhibition against *B. cinerea*, we first detected the membrane integrity of *B. cinerea* using the propidium iodide (PI) staining assay. The PI stained cells from more than 100 spores were counted under a fluorescence microscope. As shown in Table 2, compared with the control group, more spores were stained by PI and the percentage of stained cells increased in a concentration dependent manner; in the absence of BDDE, the percentage of PI stained spores was only 5.6%. However, it increased to 11%, 16%, and 23% when treated with BDDE at a concentration of 25, 50 and 100 μg/mL, respectively. These results suggested that BDDE enhanced the membrane permeabilization of *B. cinerea*.

**Table 2:** Number of total spores and PI stained spores untreated or treated with 25, 50, and 100 μg/mL of BDDE for 24 h, respectively.

BDDE (μg/mL)	Total Number of Spores	Number of PI Stained Spores	Percentage of PI Stained Spores (%)
0	123	7	5.6
25	116	12	11
50	106	17	16
100	108	25	23

### 2.5. BDDE Interacts with DNA

**Figure 5:**
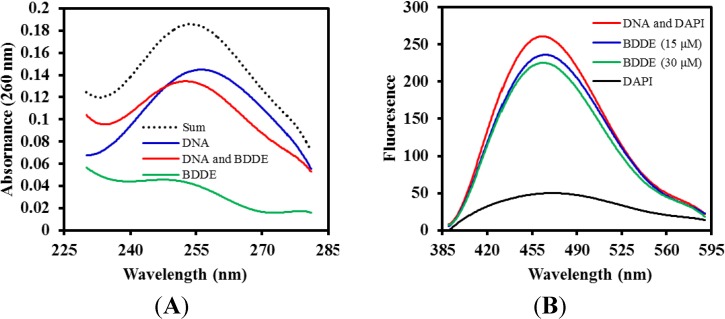
BDDE interacts with calf thymus DNA (ctDNA). (**A**) BDDE changes the absorption spectrum of ctDNA. The UV absorption spectra of ctDNA (50 μM) at 260 nm were analyzed in the presence (100 μM) and absence of BDDE using a Beckman DU 650 UV-VIS spectrophotometer (Kleve, Germany); (**B**) DAPI is displaced by BDDE as determined by fluorescence emission spectra. ctDNA (50 μM) was incubated with DAPI (1.5 μM) or concentrations of BDDE (15, 30 μM) for 30 min at 37 °C, respectively. Fluorescence emission spectra (λ_max_ = 488 nm, λ_exc_ = 340 nm) were determined using a Hitachi F-4500 fluorescence spectrophotometer (Tokyo, Japan). The experiment was repeated more than three times.

It is well established that many antifungal agents can interact with DNA molecules [19]. To investigate if BDDE could interact with DNA, a series of spectroscopic analyses was performed using calf thymus DNA (ctDNA), which is widely used to study the interaction between small molecules and DNA. As shown in Figure 5A, the UV-VIS spectrum of ctDNA displayed an obvious absorption at 260 nm, while BDDE alone only present a relatively low absorption. However, a significant suppression was found when adding BDDE to the ctDNA solution and a red shift of the maximum peak was found, indicating that an interaction happens between BDDE and ctDNA. DAPI can interact with DNA molecules by binding in the minor groove and the major groove [20]. To further confirm the interaction between DNA and BDDE, a DAPI displacement fluorescence assay was employed. The results showed that, in the absence of the BDDE, the peak fluorescent intensity of DNA and DAPI was 265 ± 16. However, the intensity decreased to 235 ± 14 and 222 ± 13, when treated with 15 and 30 μM BDDE, respectively. As shown in Figure 5B, the representative fluorescence emission spectra showed that a significant reduction in fluorescence was observed upon treatment with BDDE. The results suggested that BDDE displaced the DAPI from DNA, and there was an interaction between BDDE and DNA.

### 2.6. BDDE Intercalates ctDNA and Binds with the Minor Groove of ctDNA

**Figure 6:**
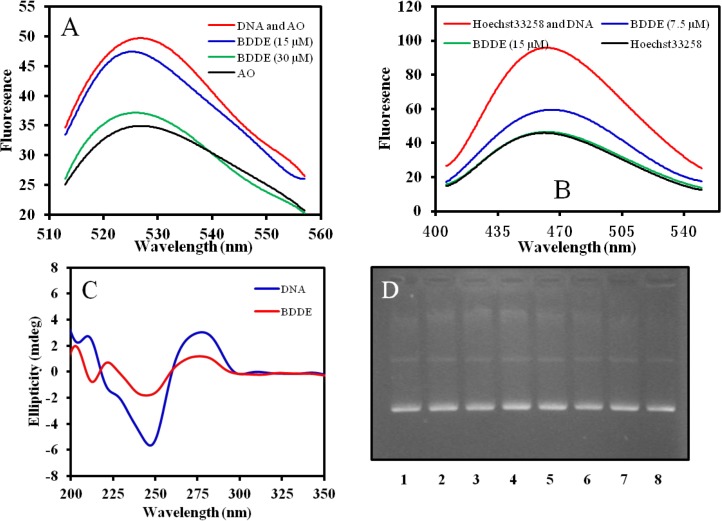
Interaction mode between BDDE and DNA. (**A**) BDDE displaces AO from DNA. AO (0.5 μM) was incubated with ctDNA (50 μM) in the absence or presence of BDDE at a concentration of 15, 30 μM for 30 min at 37 °C, respectively. Fluorescence emission spectra (λ_exc_ = 502 nm) were determined using a Hitachi F-4500 fluorescence spectrophotometer (Tokyo, Japan); (**B**) BDDE displaces Hoechst33258 from DNA. Hoechst33258 (1.5 μM) was incubated with ctDNA (50 μM) in the absence or presence of BDDE at a concentration of 7.5, 15.0 μM for 30 min at 37 °C, respectively. Fluorescence emission spectra (λ_exc_ = 352 nm) were analyzed using a Hitachi F-4500 fluorescence spectrophotometer (Tokyo, Japan); (**C**) Intrinsic CD spectra of ctDNA affected by BDDE. CD spectra of ctDNA alone (1.5 mM) or ctDNA treated with BDDE (12.5 μM) were measured with a JASCO 715 spectropolarimeter (Tokyo, Japan); (**D**) BDDE does not cleave DNA as detected by the agarose gel electrophoresis. Supercoiled plasmid pBR322 DNA was treated without (lane 1) or with 15.5 (lane 2), 31.3 (lane 3), 62.5 (lane 4), 125 (lane 5), 250 (lane 6), 500 (lane 7), and 1000 μM (lane 8) BDDE for 30 min at 37 °C, respectively. The DNA samples were resolved on 1% agarose, stained with ethidium bromide (1 μg/mL) and photographed under UV light.

Acridine orange (AO), a DNA probe agent, can intercalate into DNA molecules [21], and Hoechst33258 is a DNA binding agent, which can interact with the minor groove of DNA [22]. To further study the interaction between BDDE and DNA, displacement experiments were performed. The results showed that both AO and Hoechst33258 could be displaced from ctDNA (Figure 6A,B), indicating that the interaction between BDDE and DNA includes both intercalation mode and minor groove binding mode. The result was consistent with our previous study in that BDDE molecule fitted well into the minor groove of the DNA fragment consisting of sequential A-T base pairs [13].

Analysis of the CD spectrum also confirmed the interaction; a distinct change was found in the UV region of the CD spectrum of ctDNA when treated with BDDE, and there was a significant decrease in the positive DNA diachronic signal and a reduction in molar ellipticity in the negative band (Figure 6C). To determine if BDDE can cleave DNA directly, we incubated BDDE and pBR322 DNA together, and then checked if cleaved fragments were produced. As shown in Figure 6D, no cleaved fragments were observed, even when treated with high concentration of BDDE. These results suggested that there was interaction between DNA and BDDE *in vitro*; the interaction might include DNA binding as well as intercalation. 

## 3. Discussion

In the present study, we demonstrate that BDDE can inhibit several fungal pathogens, including *B. cinerea*, *Valsa mali*, *Fusarium graminearum*, *Coniothyrium diplodiella*, and *Colletotrichum gloeosporioides*. BDDE inhibits the growth of *B. cinerea* most clearly, and prevents the gray mold on strawberries induced by the *B. cinerea*. BDDE can suppress the spore germination and mycelial growth; treatment of the spores with BDDE results in the disruption of the integrity of the cell membrane in *B. cinerea* spores and newly formed germ tubes. Many antifungal agents inhibit the fungal growth by affecting cell membrane permeability. For example, boron decreases gray mold decay by breakdown of the cell membrane and the loss of the cytoplasmic materials [23]; dill oil shows antifungal activities by disrupting the permeability of the plasma membrane [24]. In the present experiments, BDDE induced membrane damage is observed. Loss of membrane integrity in *B. cinerea* has also been reported in several antifungal agents, including *Streptomyces globisporus* JK-1 and quinoa (*Chenopodium quinoa* Willd) alkali. These kinds of compounds usually lead to the leakage of cellular constituents, such as soluble proteins and carbohydrates from hyphae of *B. cinerea* [25,26]. Our study provides more evidence that affecting the integrity of the cell membrane is one of the main mechanisms for antifungal agents.

The results from this study reveal that BDDE can increase the cell permeability. However, compared with the inhibition rate (85%) on the mycelia growth, the percentage of PI stained spores is very low (23%), suggesting a lot of dead spores keep membrane integrity and other mechanisms may play some roles in BDDE induced fungal inhibition. Additionally, these two experiments are performed by different approaches. In the analysis of mycelia growth inhibition, the treatment time of BDDE is 2–4 days. However, in the cell membrane permeabilization experiment, the exposure time of BDDE is only 24 h before PI staining. Some antifungal agents usually result in mitochondrial dysfunction and intracellular ROS accumulation on fungal pathogens [27]. The effects of BDDE on the mitochondria and ROS need to be addressed further. Also, we have observed that on exposure of strawberries to BDDE for 10 days the antifungal effects are still present (data not shown). These primary results indicated the high stability of the compounds. However, a more systemic study is needed to address if the compound is effective for longer term preservation of strawberries or other fruits. 

In our previous report, we found that BDDE could displace EB from the DNA molecule [13]. In the present study, we show that BDDE can interact with DNA by binding in the minor groove as well as by intercalation; BDDE can also displace DAPI, AO and Hoechst33258. The data provide primary evidence that identification of compounds interacting with DNA molecules is an important strategy for finding novel antifungal agents. However, more studies are needed to address whether BDDE can interact with DNA *in vivo*; more experiments like co-localization should be performed to document the *in vivo* interaction between DNA and BDDE. 

Currently gray mold on fresh fruits is primarily controlled by application of fungicides in the pre-harvest and sulfur dioxide fumigation in the post-harvest periods, and application of these kinds of compounds usually results in problems of safety in the food supply and in the environment. Marine natural compounds, like BDDE, provide an alternate resource for the development of antifungal agents. Considering the wide antifungal spectrum of BDDE, the compound may be used in the prevention of fungal infections in different plants. Additionally, since marine bromophenols are widely distributed in marine algae and sponges, its application as anticancer, as antioxidant agents or when used as antifungal agent is worthy of further study. However, the safety of application of BDDE in agriculture needs consideration. Our previous study has shown that BDDE possess a relatively low toxicity on a non-tumorigenic epithelial cell line MCF 10A and human vascular endothelium cells (HUVEC) [13]. However, there are reports to show that some bromophenol compounds are suspected of displaying negative impact on human and animal health, acting as endocrine disruptor and moderate toxic agent on mammalian cell [4]. More studies are needed to evaluate the safety of BDDE used as an antifungal agent. 

## 4. Experimental Section

### 4.1. Drugs, Reagents, and Fruits

BDDE was synthesized as described previously [18]. Propidium iodide (PI), acridine orange (AO), 4′,6-diamidino-2-phenylindole (DAPI), and Hoechst33258 were the products of Beyond, Shanghai, China. Freshly harvested strawberries with no infection or physical injuries were bought from Tianshan orchard, Qingdao, China. 

### 4.2. Fungal Pathogens

The fungal pathogens, *B. cinerea*, *Valsa mali*, *Fusarium graminearum*, *Coniothyrium diplodiella*, *Colletotrichum gloeosporioides*, *Alternaria mali Roberts*, and *Alternaria porri* were kindly provided by College of Chemistry and Pharmaceutical Sciences, Qingdao Agricultural University, Qingdao, China. All of these fungi were cultured on potato dextrose agar (PDA) plates at 23 °C. Spores of *B. cinerea* were obtained from 14 day old cultures by washing with sterile distilled water containing 0.05% (*v/v*) Tween 80 and filtered by sterile cheesecloth. The concentration of the spores was determined using hemocytometer. 

### 4.3. Effect of BDDE on Mycelial Growth of Fungal Pathogens on PDA Plates

The effect of BDDE on mycelial growth of seven fungal pathogens was analyzed in PDA as described previously [28]. Briefly, the fungal pathogens including *B. cinerea*, *Valsa mali*, *Fusarium graminearum*, *Coniothyrium diplodiella*, *Colletotrichum gloeosporioides*, *Alternaria mali Roberts*, and *Altenaria porri* were cultured on PDA plates. After incubation for 7 days, the mycelial agar (5 mm) was cut and placed in the center of a 6-cm-diameter Petri dish containing PDA without and with 100 μg/mL of BDDE. Radial growth of each fungi was observed after incubation for 2–4 days at 23 °C and the inhibition rate of mycelial growth was calculated using the following formula: Inhibition rate of mycelial growth (%) = (1 − diameter of mycelia in the BDDE-treated medium/diameter of mycelia in the no-treatment medium) × 100. 

### 4.4. Effect of BDDE on Mycelial Growth of B. cinerea in Liquid Medium

The effect of BDDE on mycelial growth in liquid medium of *B. cinerea* was analyzed. Briefly, *B. cinerea* spores were pre-germinated in PDB medium for 24 h at 23 °C to form a germ tube. The germ tube and mycelial growth was observed using a microscope after treatment with BDDE (0–100 µg/mL) for another 24 h.

### 4.5. Inhibition of Fruit Decay by BDDE

Effect of BDDE on fruit decay was tested using the methods described previously with little modification [29]. Briefly, freshly harvested healthy strawberries were surface-sterilized in 70% ethanol for 30 s, and washed with sterile water. All of the fruits were inoculated with a *B. cinerea* spore suspension. Inocula (1.0 × 10^6^ spores/mL, 3 mL) were sprayed on about 48 berries with an air-brush sprayer. Berries were dried in air for 1 h, randomized into 4 groups (12/group), and then soaked in certain concentrations of BDDE (25, 50, 100 μg/mL) or sterile water for 10 s. After the treatment, fruits were kept in trays covered with plastic film and incubated for 5 days at 23 °C. Damage severity was observed and the decay incidence was calculated using the following formula: Disease incidence (%) = (Number of decayed berries/Total number of treated berries) × 100.

### 4.6. Effect of BDDE on Spore Germination and Germ Tube Elongation of B. cinerea

Inhibition of BDDE on spore germination and germ tube elongation of *B. cinerea* was measured as described previously with little modifications [30]. In brief, *B. cinerea* spores (final concentration of 5 × 10^5^ spores/mL) were added in PDB medium containing certain concentrations of BDDE (0–100 µg/mL) and incubated at 23 °C on a rotary shaker at 100 rpm for 12 h. Spores were considered germinated if the germ tube was equal to or greater than the diameter of the spore and a minimum of 100 spores were counted in each replicate under a microscope using a micrometer. The percentage of spore germination was calculated using the following formula: Germinated rate (%) = (Number of germinated spores/Total number of spores) × 100.

### 4.7. Analysis of Membrane Integrity Using Propidium Iodide (PI) Staining

Membrane integrity was detected using PI staining as reported previously [23]. Spores of *B. cinerea* were treated with BDDE (0–100 µg/mL) in PDB medium. After incubation at 23 °C for 24 h, spores were collected by centrifugation and stained with 10 μg/mL propidium iodide (PI) for 10 min at 30 °C. The spores were washed twice with PBS, and spread onto slides. A minimum of 100 spores were counted under the fluorescence microscopy (Zeiss, Germany). The percentage of PI-stained spores and germ tubes was calculated using the following formula: Percentage of PI negative staining (%) = (Number of PI negative stained spores/Total number of spores) × 100.

### 4.8. UV Absorption Spectroscopy

BDDE (100 μM) and DNA (50 μM) were dissolved in 50 mM phosphate buffer (pH 7.0). The absorption spectra were recorded in the presence or absence of BDDE (100 μM) using a Beckman DU 650 UV-VIS spectrometer (Kleve, Germany), respectively.

### 4.9. Circular Dichroism Spectroscopy for Secondary Structure of ctDNA

CD spectra (220–300 nm) of ctDNA (1.5 mM) treated with BDDE (12.5 μM) were measured with a JASCO 715 spectropolarimeter (JASCO, Tokyo, Japan). The spectra were collected and corrected by reduction of noise and smoothing using the program JWSSE (JASCO, Tokyo, Japan).

### 4.10. DAPI, AO, and Hoechst33258 Displacement Fluorescence Assay

DAPI, AO, and Hoechst33258 displacement fluorescence assay were employed to determine the interaction and interaction mode between BDDE and DNA. ctDNA (50 μM) was dissolved in 50 mM phosphate buffer (pH 7.0) and incubated with DAPI (1.5 μM), AO (0.5 μM), and Hoechst33258 (1.5 μM), respectively. Certain concentrations of BDDE were added and incubated for 30 min at 37 °C. Fluorescence emission spectra of DAPI, AO, and Hoechst33258 were determined using a Hitachi F-4500 fluorescence spectrophotometer (JASCO, Tokyo, Japan).

### 4.11. Agarose Gel Electrophoresis

Supercoiled plasmid pBR322 DNA (0.25 μg) was dissolved in the reaction buffer (35 mM Tris-HCl, 72 mM KCl, 5 mM MgCl_2_, 5 mM DTT, 5 mM spermidine and 0.1% BSA). Certain concentrations of BDDE (0–1000 μM) were added in the reaction buffer and incubated for 30 min at 37 °C. The reaction was terminated by lyophilizing at −80 °C and DNA samples were resolved on 1% agarose, stained with ethidium bromide (1 μg/mL) and photographed under UV light.

### 4.12. Data Analysis

Student’s *t*-test was used for statistical analysis of the data, and values were expressed as mean ± SD. Differences of *p* < 0.05 were considered statistically significant.

## 5. Conclusions

In conclusion, the present study reveals that the bromophenol BDDE displays inhibition against several fungal pathogens. BDDE decreases the germination rate, inhibits the mycelial growth of *B. cinerea*, and destroys the integrity of the fungal cell membrane. BDDE interacts with DNA via intercalation and minor groove binding, and both the membrane disruption effect and the DNA-binding activities may contribute to the antifungal effects in BDDE-induced inhibition of fungal pathogens. With the unique chemical structure different from the current fungicides, BDDE may serve as a novel antifungal candidate or parent compound for rational drug design.
